# ‘Why must I wait?’ The performance of legitimacy in a hospital emergency department

**DOI:** 10.1111/1467-9566.12072

**Published:** 2013-09-20

**Authors:** Alexandra Hillman

**Affiliations:** The ESRC Centre for Economic and Social Aspects of Genomics (CESAGen), School of Social Sciences, Cardiff University

**Keywords:** emergency departments, identity, access, individual responsibility

## Abstract

This article examines the processes of negotiation that occur between patients and medical staff over accessing emergency medical resources. The field extracts are drawn from an ethnographic study of a UK emergency department (ED) in a large, inner city teaching hospital. The article focuses on the triage system for patient prioritisation as the first point of access to the ED. The processes of categorising patients for priority of treatment and care provide staff with the opportunities to maintain control over what defines the ED as a service, as types of work and as particular kinds of patients. Patients and relatives are implicated in this categorical work in the course of interactions with staff as they provide reasons and justifications for their attendance. Their success in legitimising their claim to treatment depends upon self-presentation and identity work that (re)produces individual responsibility as a dominant moral order. The extent to which people attending the ED can successfully perform as legitimate is shown to contribute to their placement into positive or negative staff-constituted patient categories, thus shaping their access to the resources of emergency medicine and their experience of care.

## Introduction

This article provides an account of the negotiations that occur during patient assessments in an emergency department (ED) and shows how patients and their relatives help determine their placement into positive or negative patient categories as constituted by the staff. There is a strong tradition in medical sociology of examining the categorisation and ordering work undertaken by medical staff in prioritising patients for treatment and care (Dingwall and Murray 1983, Dodier and Camus 1998, Hillman 2008, Hughes 1976, 1980, 1988, 1989, Jeffrey 1979, Latimer 1999, Mannon 1976, Roth 1971, 1972a, 1972b, Roth and Douglas 1983). This body of work challenges the assumption that the ordering of patients into categories of priority is solely a clinical endeavour.

Categorisation work attempts to identify the legitimacy of patients’ claims according to interconnecting social, organisational and clinical criteria. Such legitimisations tend to be predicated on the types of cases that patients are determined to be (Roth 1971). Hughes (1976) shows how professional clinical practice is accomplished through processes of practical decision-making embedded in and dependent for their significance upon knowledge thought of as common sense. As in all walks of life, clinical decision-making depends upon drawing on typifications, conceptions based on the type of case staff perceive the patient to represent. For staff working in the ED, this involves a continuous task of ‘making their environment meaningful and intelligible by ordering their experiences in terms of familiar categories’ (Hughes 1976: 138). In the case of the ED, these typifications are built upon and added to by a variety of staff both professional and non-professional over the course of the patient’s journey (Hughes 1980, 1989, Mannon 1992).

EDs are significant settings for this kind of categorisation work due to their position within the wider health service. Firstly, they are arguably the most open service existing at a threshold between public in need and hospital care. Secondly, they function as gatekeepers controlling access to acute care through the admission of patients to acute hospital wards. The ED is a site of potential conflict between the valued work of emergency medicine – constituted as expert medical intervention with fast, measurable results – and the needs and demands of those who attempt to gain entry. This conflict ensures that certain kinds of patients represent for staff an ‘exercise in futility’ (Mannon 1976: 1004), drawing them away from the core purpose of ED work. This potential conflict manifests in the ED through the ways in which patients are sorted out by staff into categories of need according to complex medical, administrative and sociocultural systems of classification. This article focuses specifically on triaging, a process that most overtly relies on practices of categorisation. As Gibson (1978) notes, it is at the triaging stage of the ED that staff evaluate cases on the basis of medical and moral criteria and ‘it is at this stage that most negotiation will occur, where the appropriateness of the injury will be established’ (pp. 16–17). These processes also provide staff with the means by which to gain control over what they determine to be inappropriate demands for the service (Roth 1972a).

Two overarching themes that emerge from this literature have particular relevance to the contemporary categorising of patients for priority of care and treatment in the ED: firstly, the categorisation of patients according to the professional interests of clinicians and the broader organisational structures and institutional cultures of medical practice and secondly, ideas of personhood and the perceived moral worth of patients and their families.

Moral evaluation is founded upon the application of concepts of social worth common in the larger society (Roth 1972a). They draw on staff’s experiences of social life and their capacity to typify these experiences to enable judgements regarding the types of cases their patients represent. It is therefore unsurprising that individual responsibility, moral worth and medical practice are connected in ways that have interested sociologists beyond the field of medical sociology. Parsons’ (1951) work on the social system, for example, argued that every situation in which a person claims illness implies a responsibility of the patient for the disease. Parson’s model highlighted the rights and responsibilities attributable to the sick person. Individuals classified as ill are exempted from the duty to abide by normal social role obligations. However, this partial exemption is conditional on certain responsibilities being met, such as the recognition of sickness as an undesirable state and the need to seek help for, and cooperate in, the process of getting better. As a consequence, no illness or condition is intrinsically guilt free. Instead, the acquisition of guiltlessness depends upon an implicit contract between the doctor and patient. Those who break the rules that govern this contract over who is awarded legitimate entry into the sick role are, in Parsons’s view, regarded as deviant and liable to punishment. For Jeffrey’s (1979) study of an emergency department, this punishment manifests itself through patients being left to wait for longer periods of time, the delaying of their treatment or, in the case of a ‘self-harmer’, being left ‘in the hope they’ll get annoyed and take their own discharge’ (p. 103).

McHugh’s (1970) work on deviance has also informed medical sociology’s interest in processes of patient categorisation. Both Jeffrey (1979) and, to a greater extent, Dingwall and Murray (1983) draw on McHugh’s analysis of the social accomplishment of deviance to inform their interpretations. McHugh identifies two key attributes necessary for deviance to be socially recognised. Firstly, deviant behaviour must have conventionality. This refers to the extent to which an act is deemed to be out of the ordinary, an act that ‘might not have been or might have been otherwise’ (p. 61). Secondly, there must be theoreticity. This refers to the agency of the actor or the extent to which they know what they are doing. The behaviour alone, according to McHugh, does not provide other members of the group with all the necessary clues to determine deviance. McHugh (1970) uses the example of children as a key indicator of the significance of theoreticity, noting that the same behaviour in adults and children may be treated very differently; an argument developed by Dingwall and Murray (1983) in their analysis of children’s treatment in an ED.

In this analysis the relationship between deviance and illness differs from Parson’s sick role in that illness itself is not necessarily deviant, but is instead morally ambiguous. Sick individuals remain vulnerable to charges of deviance, but the resolution of those charges depends on a process of negotiation. This distinction is particularly useful for the categorisation work described in this article, which directly focuses on these processes of negotiation and shows how patients and relatives are implicated in staff’s production of their illness behaviour as either deviant or legitimate, as attendees’ accounts are produced in the knowledge that they will be scrutinised for their moral content.

The body of work that predates this contribution illustrates how staff make sense of the previously unknown patients they are faced with in the ED environment. This study provides new insights by showing the ways in which patients and relatives are drawn into the co-production of the patient categories used to order ED work. Staff categorise their patients according to the type of case a patient represents. These types are constituted according to the organisational and institutional interests of the department and the interests of clinicians themselves. However, the fixing of such categories to patients is negotiated in the interactional processes that occur between staff, patients and their families. Roth’s (1972a) study, in particular, established the reciprocal nature of the relationship between patients and their (perceived) attributes and staff-constituted patient categories. Furthermore, he identified this relationship as the basis upon which moral judgements are made. This article focuses specifically on the nature of this relationship to show how the identity work of patients and their relatives has significant consequences for determining the patients’ placement into staff-constituted patient categories and subsequently their access to and treatment in the ED service.

## Methods and methodology

This ethnographic study of emergency medicine was carried out in a large inner city UK teaching hospital with a particular focus on the assessment, care and treatment of older people. The project ran over 4 years between 2004 and 2008 and received ethical approval from a National Health Service (NHS) national research ethics service committee and research governance approval from the NHS trust involved. The site was chosen on the basis that it represented some core characteristics of large, inner city ED services. Firstly, it is a university teaching hospital and as such presents itself as a centre for excellence (Vetter 1995). Secondly, it serves a large and diverse population; a population that has increased as a result of the closure of smaller, community based emergency services. This is a phenomenon mirrored across the UK.

Participants in the study included medical staff of all levels from healthcare assistants to the clinical director and patients, patients’ relatives or carers and managerial staff. Observations were carried out across distinct areas of patient care within the ED and visits were arranged to cover the seasons, the days of the week and times of the day and night. The observations were flexible and unstructured but loosely took two approaches: either patients were tracked from their initial assessment to their eventual admission or discharge or members of staff were shadowed while working their shifts. The meanings of the actions and interactions observed were further elicited and explored through formal and informal interviews with staff and patients.

The 250 hours of observation accumulated over the course of the study included the tracking of 50 older patients (over 65 years of age) through their assessment in the ED, and 15 of those older people were interviewed following their assessment. The 50 older patients tracked through the department were selected on the basis of their age and their willingness and ability to consent to take part in the study. Although the focus of the study was on the experiences of older people, the assessment and treatment of patients under 65 years of age became a central part of understanding the culture of the ED and older people’s place within it.

The observations of patient assessments focused on how staff accomplished categorising patients for priority of treatment, the negotiations that occurred during these clinical encounters and the meanings of these interactions for staff and patients and their relatives. The observations also paid attention to the organisational cultures of emergency medicine to situate these processes of assessment in their broader context.

Both field notes and interview transcripts were analysed thematically and the transcripts and field notes were simultaneously analysed while carrying out fieldwork. This meant that emerging issues, such as the categorising of patients for priority of treatment, could be read and interpreted alongside broader institutional concerns of throughput and the rationalisation of resources.

The researcher’s position as observer was reflected upon to identify influences on the encounters observed. The researcher was sensitive to the vulnerabilities of participants and always ensured both staff and patients were aware of their voluntary participation. Decisions over when to observe, how to stay attuned to the wishes of those being observed and when to withdraw altogether were continually negotiated in the field between the researcher, the patient and staff participants. This approach to the process of obtaining and maintaining consent from research participants – that recognises consent as a process of continual negotiation – reflects the nature of qualitative research whereby not all potential ethical dilemmas can be anticipated at the outset (Renold *et al*. 2008). These ethical decisions, as well as the role the researcher played in the encounters they observed, were recorded in the field notes and informed the interpretation of the data.

The data collected together within themes were checked for the consistency and validity of interpretation. The constant comparative method (Glaser and Strauss 1967) was used to check the relationship between concepts and to build common themes. Initial analysis was discussed with practitioners and patients informally during fieldwork visits as a means of respondent validation (Bloor 1978), ensuring that project themes resonated with the experiences of those in the field.

The examples presented in this article are taken from the field notes of observations. The examples mainly draw on the assessments of older people although they are not restricted to this group. All the patients in the field note extracts happen to be women. This is not to suggest that men do not partake in practices of negotiation during their assessments. Rather, the examples presented were chosen as they best represent the key strategies employed by patients and relatives to aid their entry into positive categories constituted by the staff as appropriate or legitimate. The observations of these encounters and the conversations and accounts that followed occurred in the triage unit of the ED.

The following three sections present examples of categorisation work and illustrate how staff-constituted patient categories are co-produced in the interactions and negotiations that occur between staff, patients and patients’ families.

## Accessing the ED, identity work and the problem of passing

The triage system is set up to prioritise patients according to clinical need. It is managed from within the minor injuries unit. As the previous discussion of the literature illustrates, practices of categorising patients according to priority cannot be dependent upon asocial, clinical truths. The ED’s triage system is no different. Simple organisational issues such as the time of day or night that a patient arrives at the department can mean the difference between placement into one category or another depending upon how many other patients have been triaged into categories of more or less urgency. The point at which a patient is seen in the course of a member of staff’s shift (see Dingwall and Murray 1983) can also shape clinical decision-making. The usefulness of a particular patient in enabling staff to show their competencies, aid their professional development (Jeffrey 1979, Latimer 1999) or help (re)accomplish emergency medicine as a specific acute specialty, also informs the decision-making process in which patients are allocated to a particular triage category.

The triage unit is therefore a space in which staff are best able to perform a ‘labour of division’ (Hetherington and Munro 1997), sorting patients out into categories of good, interesting/normal rubbish, legitimate/illegitimate and appropriate/inappropriate (Dingwall and Murray 1983, Jeffrey 1979). The act of assigning patients to a triage category is carried out by triage nurses working in the minor injuries section of the ED. However, as the examples presented in this article show, the process of categorisation involves a number of staff that can include reception clerks, paramedics, nurses and doctors. As Hughes (1980, 1989) makes clear, typifications tend to emerge and become elaborated, revised or rejected both over time and through the interactions of various staff members who account for the patient’s condition. Furthermore, the interactions between staff that shape patient categorisations can transcend traditional staff hierarchies (Hughes 1988).

**Figure 1:**
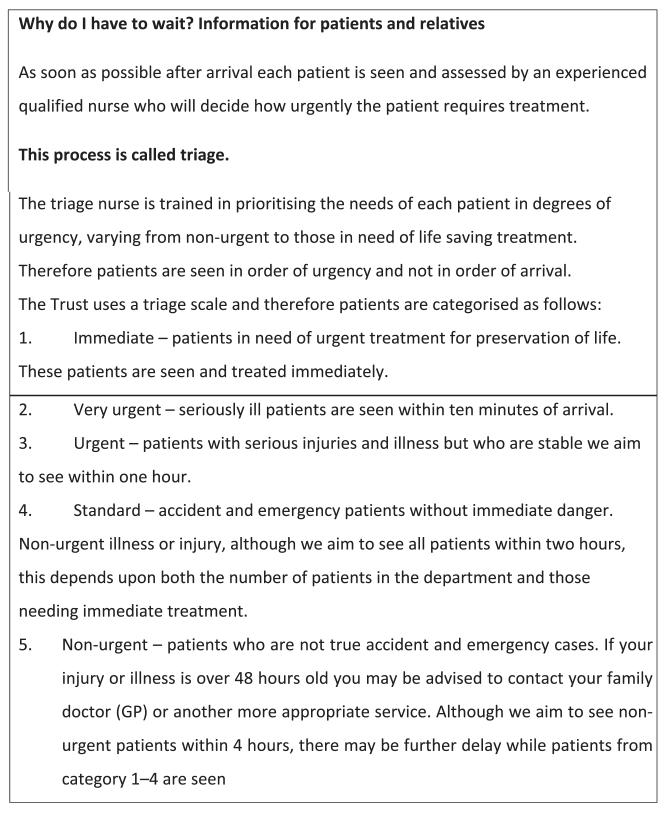
Why do I have to wait? Information for patients and relatives

Patients are made aware that decisions about how long they will be left to wait are determined according to the triage system. A leaflet entitled ‘Why do I have to wait?’ provides patients with information about the system and is placed on waiting-room chairs. The content of the leaflet is displayed in Figure 1.

The examples in this article refer to patients who were categorised into triage categories 4 and 5. This reflects most of the cases assessed in the ED. Common complaints such as fractured ankles, small cuts and mild concussions make up most of the triage nurses’ workload. It was also during decision-making in relation to the allocation of patients to categories 4 and 5 that patients’ own accounts were most overtly implicated. It is not the author’s intention to suggest that the sorts of categorisation work described were not implicated in decisions regarding the allocation of patients into categories 1, 2 or 3, or that these categories were somehow immune from sociocultural forms of classification. The consequences of being categorised as triage category 4 or 5 are significant for patients. As will become clear, the allocation of patients to triage categories is in part a mechanism through which staff are able to reward or punish patients on the basis of their perceived appropriateness to the ED service.

The following extract tells the story of Mrs Johnson’s visit to the ED. The key aspect of her self-presentation is her denial of rights to services.

Mrs Johnson: ‘an oldie but goodie’

Later that morning, two paramedics arrived at the door of assessment room one with a patient assessment form for an 89-year-old woman who they’d brought in with a fractured foot. They both raved about how lovely she was and how she had fallen a week earlier. One of them explained to the nurse that she ‘didn’t want to bother anyone, you know’. Nurse Morris turned to me and said ‘There you are you see’, quite pleased that Mrs Johnson had proven her point that elderly patients tended not to use EDs unnecessarily. The paramedics then wheeled Mrs Johnson in on a chair for Nurse Morris to assess. Mrs Johnson was clearly very frail, but she was also very well turned out, neatly dressed and her hair was combed and tied neatly at her neck. Nurse Morris smiled, said hello and asked her how she was feeling:

Mrs Johnson I’m 90 tomorrow so I can’t complain; I’m in good health really.

Nurse Morris So what happened to you? Why didn’t you come in and see us sooner?

Mrs Johnson Well I see on the news about how terrible it is here and I err … I didn’t want to take up space you see … it’s just that I’m living alone that’s the trouble you see.

Mrs Johnson is well spoken and pleasant to the medical staff. She has a calm and friendly demeanour and abides by the correct rules of patient behaviour (Glaser and Strauss 1970). Interestingly, other patients who had suffered pain for long periods of time before arriving at the ED were reprimanded for not seeking advice from their general practitioner (GP) sooner. As one nurse explained to me, a person suffering pain for a long period of time is no longer deemed the sufferer of an accident or an emergency. The temporal nature of a patient’s complaint is a longstanding concern of ED staff. Gibson’s (1977) doctoral study describes the similar significance placed on the length of time a patient had suffered their presenting ailment in assessing their eligibility. Mrs Johnson was therefore vulnerable to the charge of deviance, for not seeking the correct help and support for her ailment and not taking action to get better, to draw on Parsons’ (1951) model. However, Mrs Johnson negotiates her placement into a positive staff-constituted category of legitimacy through her own reasoning. This is partly accomplished through the context of the clinical encounter itself. The contexts and contingencies of each clinical encounter shape the content of that interaction, including the patient’s story and the staff’s response. Mrs Johnson’s account does not reflect an enduring feature of her personality; her story is a reflection of the clinical encounter (Clark and Mishler 1992). In this circumstance, the paramedics had already responded positively to elements of Mrs Johnson’s story that described her attempts to avoid burdening the ED service. She has, as a result, some resources with which to present herself more positively in her interactions with Nurse Morris.

She explains, she ‘didn’t want to bother anyone’. This is partly due to her experience of watching the news about ‘how terrible it is’ reflecting the discourse of crisis that surrounds NHS provision and EDs in particular. For those patients who have not experienced the crisis discourse that Mrs Johnson describes seeing on the news, there are many methods of incitement through which patients are made to understand the necessity of accounting for themselves once they arrive at the ED (Hillman 2008). These incitements call upon patients to provide a particular kind of self-presentation similar to that captured in Garfinkel’s (1967) concept of ‘passing’. The patients must undertake the work of achieving and making secure their entry into the category of legitimate patient while providing for the possibilities of detection or recognition as a deviant. This possibility is an ever-present risk within the socially structured conditions of the ED service, in which patients’ legitimacy is never assumed. It is clear that Mrs Johnson recognises the possibility of her potential charge of deviance through the reasons she provides for not seeking help earlier: she didn’t want to ‘bother anyone’, a description that fits the definition of the situation (Goffman 1959) – a definition carefully maintained by those working in the ED – as a service in crisis.

Mrs Johnson’s comment that she did not want to take up space suggests she perceives her claim to the services of emergency medicine as being less legitimate than claims made by others. This perhaps reflects the discourses that surround older people and the provision of health services. Older people’s attendance at emergency departments is often cited as one of the major factors in accounting for the so-called crisis of the NHS, being labelled as bed blockers, socials or acopias by clinical staff and health service managers (Hillman 2008, Latimer 2000, Oliver 2008). It is Mrs Johnson’s presentation of her responsibility in relation to accessing health services that aids her case with Nurse Morris and the paramedics.

As a consequence, Mrs Johnson is rewarded. The paramedics bring her directly to the triage nurse rather than being shown to the waiting area, as would normally be the case. This example shows that a significant skill for passing as a legitimate ED patient – in providing good reasons and justifications for accessing the ED – is to deny expectations of care. Instead, an understanding of a moral duty to be responsible for one’s health and wellbeing must be demonstrated. This is significant for the perception of the individual patient’s worth and the perceived legitimacy of their claims to emergency health resources.

In order for patients and relatives to account for their responsibilities in relation to the accessing of public resources, there must be an understanding of which services should be approached, when and by whom, as is illustrated in the following extract below:

It’s not their fault so I’ve put a category four

Nurse Price takes me with him to assessment room 2 where he was triaging Jesse, an 89-year-old woman who had been brought in by her daughter, Valerie. Jesse is in a wheelchair and looks frail and withdrawn. Valerie pulls Jesse in through the door and takes a seat next to her and starts explaining to Nurse Price what the problem is:

Valerie She’s had a bit of a fall out of bed in the night, my sister called the ambulance because the GP refused to come out and see her. I think she panicked to be honest; we’re probably wasting everybody’s time …. Her wee is a little smelly too … the GP had said she needed another catheter but she’s been passing water fine.

Nurse Price There’s no need for that, it’s most likely she’s just got a urinary infection. [Raising his voice] Jesse, what is your date of birth, can you tell me?

Jesse [straining to hear] Oh um its err 16 of March, 1916.

Valerie She is a little confused: not bad on long-term memory but her short-term memory isn’t so good.

Nurse Price Okay, that’s fine. If I just give you this [hands Valerie a pot for a urine sample] so that we can check your mum’s urine. The ladies toilets are just back out onto the corridor and on your left. If you could just pop it back in to us when you’re done and we’ll get the doctor to call you back once we’re ready for you, okay?

Once Valerie and Jesse had left the room, Nurse Price turns to me and says:

Classic case of they shouldn’t be here but it’s not their fault. It’s outrageous that the GP wouldn’t see them. Really this should be a category 5 case but it’s not their fault so I’ve put a category 4.

Jesse is frail, confused and has difficulty hearing. Almost immediately Nurse Price elects to speak to Valerie (Jesse’s daughter) rather than the patient herself. Jesse is a good example of a patient who may have been categorised by staff as another ‘social’, whose social circumstances (her age) obscure her clinical needs rendering her inappropriate for the ED (Latimer 2000). As Nurse Price comments, ‘they shouldn’t be here’. McHugh’s (1970) understanding of deviance is particularly useful in understanding Jesse’s case. The illness presented by Jesse and Valerie to Nurse Price is not in itself seen to be appropriate to the ED. Their action in attending the ED could have been (or should have been) otherwise. In other words, their actions meet McHugh’s (1970) description of conventionality in determining and recognising deviant behaviour.

However, Valerie gives an account of their reasons for attendance and attends to the issue of fault or responsibility. Firstly, as in the case of Mrs Johnson, there is no assumption during her account that they will and should receive access to these services. Daughter and mother refrain from claiming entitlement to treatment and Valerie’s story highlights the circumstances that have led them to present even when ‘we’re probably wasting everybody’s time’. This, as with Mrs Johnson, attends to the problem of passing (Garfinkel 1967) that necessitates that patients (and relatives) perform according to the given definition of the situation (Goffman 1959) in which patient legitimacy must be negotiated and is never assumed.

Unlike that of Mrs Johnson, in Jesse’s case it is through making it clear that they have attempted to see the GP before attending the ED that ensures their success in gaining access. It is this part of Valerie’s story that attends to the issue of agency. She suggests that they had no choice in making the decision to attend the ED, shifting the responsibility for the deviant act and failing to accomplish McHugh’s secondary necessary attribute for accomplishing social deviance, theoreticity. In the performance of correct service use, accomplished through Valerie’s account that the most appropriate route through which to see a doctor had failed, the responsibility for inappropriate attendance shifts from being that of Jesse or Valerie and becomes the fault of the GP and, more generally, the primary-care sector.

Categorisation on this basis is done as part of a wider notion of responsibility, the responsibility patients as consumers of health services (Sointu 2005) have to manage their own health needs. The response to this legitimisation is to reward Jesse and Valerie to a higher triage category than the perception of the patients’ clinical condition may otherwise have warranted. This reward manifests itself as a shorter waiting time, a doctor’s assessment and the testing of a urine sample, services that may have been denied to the patient, if she had been placed in triage category 5 where patients ‘generally don’t get seen, we just let them wait for hours in the hope that they’ll go home’.

## Disciplining inappropriate patients

If good reasons and justifications are not made to pass successfully as a responsible health service user, patients can find themselves the subject of various forms of discipline. This disciplining not only has significant consequences for accessing hospital services but also for regulating patients so that they are made aware of their responsibilities in the future. They are imparted with the knowledge of what determines a responsible healthcare user. This is not only seen in the interactions between staff and patients: more subtle forms of disciplining that attempt to inform patients and incite them to perform their responsibilities are also evident.

The leaflet entitled ‘Why must I wait’ (Figure 1) is a good example of this disciplining. The function of this leaflet is not only to appease patients who are frustrated by how long they have to wait or by seeing people who may have arrived after them being seen before them, it also works to regulate those patients who may fall into triage category 5. This leaflet is not merely providing information; there is an attempt to discipline those who are ‘not true accident and emergency cases’ by warning that their treatment may be delayed or they may be referred elsewhere and, conceivably, encouraging some attendees not to wait for assessment at all.

The leaflet is also a means by which to maintain the definition of the situation (Goffman 1959). In highlighting the need to prioritise, the leaflet helps (re)establish the ED as a service in crisis. Such a definition of the situation ensures the potential detection of patients as deviant is felt and the subsequent work is undertaken to secure their passing into a category of legitimacy (Garfinkel 1967). Perhaps those who do read the leaflet are more likely to demonstrate good reasons and justifications for attending the ED. This article shows that in part, the system of triage is available as a means through which to discipline and reward patients on the basis of their presentation as health responsible individuals.

The extract below describes a more explicit form of disciplining that occurs for those patients who, according to the staff, should not have attended the ED at all:

Room 8 and clinical solutions

It was during this visit that I discovered Room 8. Two patients were sent to Room 8 in the space of an hour towards the end of my evening at the emergency department. Room 8, as it is explained to patients, is for ‘clinical solutions’. It is for those patients who present at the ED with problems that are deemed to be more appropriate to primary care. Room 8 simply consists of a phone where the patient is able to call the GP out-of-hours service.

I heard Sister Smith talking about the first patient who was sent to Room 8. I had not seen her being assessed but from Sister Smith’s conversation with Nurse Harbury, the patient was a young woman who had come in to the department with suspected cystitis which, as the sister points out, ‘is not an ED problem’.

The second young woman, who was in her early twenties, was suffering from what seemed to be ‘flu like symptoms. During her initial examination and assessment with Sister Smith she had told her that she was generally fit and well. Following this initial assessment and brief history, the nurse sent her to Room 8 to contact her GP. Later, the woman came back to the assessment room to see Sister Smith to explain that the practice nurse at her GP’s surgery had told her to come to the ED and ask for the HIV consultant. She told her that she is HIV-positive and that the nurse at her GP surgery had felt that her symptoms were serious because she also suffers from a heart defect. Sister Smith tells the woman to go back outside and wait. Once the girl had left the assessment room, she turns to Nurse Harbury and says ‘I can’t understand why she didn’t tell me that in the first place. Well if she feels that she should be seen by the consultant, she will have to be referred by the GP’.

The GP out-of-hours service provides patients with advice or an appointment at a primary care service, normally with the patient’s own GP. Essentially, the responsibility for the patient’s problem is shifted onto the primary care sector. Interestingly, Room 8 could be seen as a method for turning patients away, a practice that is illegal in other countries including France (see Vassy’s 2001 study of a French emergency department). However, Room 8 is specifically for patients identified as not true A&E cases. From the point of view of the staff and the organisation, these patients do not count within the rules of the system. Such patients might also wait longer than the 4-hour target, which stipulates that all patients in the UK must be seen within 4 hours of being triaged. But from the standpoint of staff this would not constitute a breach of the target as these individuals fall outside the official definition of ED patients.

In Sister Smith’s conversation of the first case to be sent to Room 8 there seemed to be no hesitation that this was the correct course of action to take. Cystitis was firmly in the category of ‘not an ED problem’. In this case, just as the leaflet on the waiting-room chair had hinted, there is a need to discipline the patient in order to encourage behaviour that better demonstrates an understanding of the moral duty to use services appropriately.

For Claire, the young woman who is HIV-positive, the risks of serious ill health seem to be higher than would normally warrant sending a patient to Room 8. It is rather the account that Claire provides that merits this discipline. She fails to perform according to the definition of the situation, in which legitimacy must be secured through the patient’s own proffered reasons and justifications in order to militate against a charge of deviance. She fails to demonstrate her responsibility for managing her illness through using the most appropriate services for her needs at the correct time. Unlike Valerie, she does not demonstrate a knowledge of the system in which there are set routes that must be followed in order to gain access to services such as a consultant examination. As a result, Claire gets sent to Room 8 and must go back to her GP before accessing the assessment she requires.

By returning to McHugh’s (1970) concept of deviance once more, it is possible to understand Claire’s case in the opposite way to that presented by Jesse in the previous example. Unlike Jesse, her illness and the subsequent attendance at ED itself is not automatically understood as deviant. There are risks associated with the patient’s presenting history and she has been advised by a health professional to attend. Whether the conventionality component necessary to attribute deviance is present in Claire’s case is therefore ambiguous. It is the theoreticity, the extent to which Claire is thought to know what she is doing, that ensures her placement into a deviant category. The route through which Claire attempts to gain access to an assessment by a consultant suggests that she is knowingly accessing services inappropriately because she is holding back necessary information. Furthermore she does nothing to attempt to shift responsibility. As Sister Smith notes, ‘if she wants to be seen by a consultant, she must get a GP referral’. This is significant. For Claire to seek this alternative route indicates a perceived entitlement and fails to demonstrate her responsibility to reduce burdening a stretched health service. She does not perform to the definition of the situation. Finally, the perceived deception with which Claire had presented her reasons for her attendance further contributes to her problematic presentation. By initially concealing her illness, there is not only a resultant lack of trust between herself and Sister Smith but also the suggestion of that she is unable to appropriately manage her health needs. Had Claire performed the sort of self-responsible conduct (Sointu 2005) of patients such as Mrs Johnson or Valerie she may have experienced a more successful negotiation and would not have found herself the subject of disciplinary action.

## Discussion

The contribution of this study is to identify the consequences of patients’ and relatives’ own accounts for determining their placement into staff-constituted patient categories. Patients are incited to justify and provide reasonable explanations for their attendance during their negotiations with clinicians in order to pass as legitimate. Patients who may otherwise have been labelled negatively by staff according to staff’s own means of sense-making, the typification of older patients as ‘socials,’ for example, are shown to shift the responsibility for their inappropriate attendance and thus ensure their placement into a more positive staff-constituted patient category. Patients are not generally fixed into categories of good or bad, an over simplistic dichotomy that has resulted in criticism of Jeffrey’s (1979) categories of good or interesting patients and normal rubbish. Instead, a patient’s status is more fluid and continually in the making. It remains the case that certain patients, including older people, are more vulnerable to negative labelling. However, this study highlights the strategies of negotiation that can enable patients to avoid a charge of deviance and pass as legitimate.

The potential for a charge of deviance resulting from inappropriately accessing a service ‘in crisis’ is accomplished through the socially structured conditions of the ED. Part of these conditions includes the corporatisation of NHS hospital services. Staff working in the ED must manage the patients as a group making claims to the resources of time, treatments and examinations. This management task manifests itself during processes of audit and self-checking for patient waiting times, discharge rates and the number and cost of examinations and treatments. Such monitoring systems ensure that the institutional concerns of demand management, accountability and risk (see Hillman *et al*. 2013) are embedded in everyday clinical work. Ideas of appropriateness therefore reflect and reproduce the institutional interests of contemporary health services. Previous work (Griffiths and Hughes 1994) has shown how wider systems of healthcare rationalisation shape the nature and extent to which clinical staff draw upon ideas of morality in the categorisation of their patients. In the context of the ED, ideas of appropriateness and the processes through which staff attribute legitimacy or deviance are shaped by the increasing rationalisation of the ED as a service to treat strictly acute trauma cases (Hillman 2008). Such processes reflect a changing ideological landscape of the NHS in which affordability and rationalisation in relation to treatment access are overriding the traditional NHS values of universality and comprehensiveness (Russell and Greenhalgh 2012).

Patients and staff’s co-production of appropriateness in their negotiations over access to the services of the emergency department bring to light a cultural shift in the relationship between individuals and health services. There is a growing emphasis in the UK and in other western societies on self-responsible conduct (Sointu 2005), whereby patients are expected to show awareness of their personal obligation to manage their health and wellbeing. Individuals are increasingly required to self-regulate and manage their personal health risks as part of a moral order that views individual regulation of health risks as paramount to the alleviation of public services in crisis. The shift in health responsibility from the state to the citizen is becoming increasingly common, particularly in primary care in the UK (McDonald *et al*. 2007). This study shows that the ED, as a space between hospitals and primary care, is becoming a place in which patients are incited to demonstrate their appropriateness and, for those who fail, are imparted with the knowledge with which to display morally responsible service use in the future.

Greco (1993) suggests that, in our time, the implied responsibility of patients for their illnesses, outlined in Parsons’ (1951) work on the sick role, has become explicit and amenable to some kind of rational decision-making. In part this has resulted from the media being able to make this responsibility collectively visible and conscious, something that medicine has been unable to achieve for each individual. Mrs Johnson’s concerns about the news stories she had seen on television is a good example of the way in which rational choice about, and responsibility for, individual health care have become integral to wider understandings of illness.

The ED can therefore be understood as a site in which the responsibilities of a health service in crisis are increasingly being individualised. The individualisation of societal problems is a pattern being replicated across the western world both in relation to health (Asquith 2009) and the environment (Butler 2010). People’s troubles and misfortunes are understood as the inadequacy of the self rather than being dependent upon conditions outside the realm of an individual’s control (Bauman 2001). What is obscured as a result of these dominant discourses is an appreciation that health need, and our responses to it, reflect our social and collective life. In other words, there is a contradiction between the attribution of individual responsibilities and tacit acceptance of societal systems and cultures that promote health inequalities and sustain so-called risky health behaviour.

This research shows that the contemporary ED is where the work of accomplishing blamelessness is carried out. It is a place in which the moral ambiguity of illness is worked out on the basis of patients’ own accounts. McHugh’s (1970) idea of theoreticity is particularly helpful in highlighting the necessity of such negotiations in reaching a charge of deviance or legitimacy. Furthermore, the examples from this study show the precariousness of these interactions for patients and relatives. The identity work necessary to pass as an appropriate ED patient is not easily accomplished and the threat of being charged with deviance is ever-present.

The implications for those unable to provide persuasive accounts and pass as legitimate are significant. If it has become the responsibility of individuals to secure themselves against the risks that ill health pose to health services, the emphasis is placed not on a person’s absolute need but on their duty to justify their need (Joyce 2001). Those patients who are not able to successfully justify their need suffer the consequences of being placed in negative, staff-constituted patient categories. This can mean the refusal of treatment either through being sent to Room 8 or through being placed in triage category 5 and being left to wait indefinitely, the delay of examinations, tests or treatments or a lack of care and compassion from ED staff. As well as acting as barriers to health services, these forms of discipline have important consequences for patients’ experiences of care. Being left to wait indefinitely can leave patients feeling hopeless. Furthermore, the public refusal of access to emergency services works to re-affirm patients’ diminished expectations and makes clear that the receipt of care from public institutions is no longer given without patients having to work hard to justify their need.
